# Investigation of moral intelligence’s predictive components in students of Shahid Beheshti university of medical sciences (SBMU)

**DOI:** 10.18502/jmehm.v13i13.4389

**Published:** 2020-09-20

**Authors:** Maryam Mohammadi, Shabnam Mohammadi, Ali Mehri, Fatemeh Bagheri Mazraeh

**Affiliations:** 1 *Assistant Professor, Health Education and Health Promotion, Department of Public Health, School of Public Health, Shahid Beheshti University of Medical Sciences, Tehran, Iran.*; 2 *Assistant Professor, Neurogenic Inflammation Research Center, Mashhad University of Medical Sciences, Mashhad, Iran.*; 3 *Assistant Professor, Department of Health Education, School of Health, Sabzevar University of Medical Sciences, Sabzevar, Iran.*; 4 *Researcher, Student of Public Health, School of Public Health, Shahid Beheshti University of Medical Sciences, Tehran, Iran.*

**Keywords:** Moral intelligence, Students, Medical sciences, Lennick and Kiel’s model.

## Abstract

This study aimed to investigate dominant predictor components of moral intelligence (MI) based on the Lennick and Kiel's model in students of Shahid Beheshti University of Medical Sciences (SBMU).

In this descriptive-analytical study, 322 students of SBMU were chosen through cluster sampling. To collect data, a 40-item questionnaire, whose validity and reliability was confirmed in previous studies, based on the Lennick and Kiel's model was used. The collected data were analyzed by SPSS 21 software using appropriate descriptive and analytical statistics. Of 322 participants, 180 and 142 were female and male, respectively. The mean age of the participants was 22.30±2.69 years. The study’s regression analysis revealed that the most and the least direct effects were related to the forgiveness (R2=0.320) and compassion (R2=0.284) components, respectively. Among the inspected components, the responsibility component with an overall effect of R2=0.655 was shown to be the strongest predictor component of MI. Universities play a significant role in students’ moral development and enhancement. The present study’s findings suggest that developing strategic plans and interventions can enhance MI level (e.g., incentive systems for individuals maintaining high moral responsibility). Since today’s students will be tomorrow’s medical and healthcare professionals, upgrading of MI level in students studying in various divisions of medical sciences enhances their moral responsibility through setting out strong ethics principles to follow and the quality of care that they will provide to patients, thereby improving health.

## Introduction

Moral Intelligence (MI) can be referred to as human’s capacity to distinguish right from wrong and to apply moral principles to humans’ intentions, goals, beliefs, values, and actions. As a newly- introduced concept, MI has been less researched compared to other types of intelligence (e.g., social, emotional, and cognitive). Recently, MI has received more attention in researches conducted in psychology and management fields (1). MI provides a framework for humans to act in accordance with moral principles; such framework can provide potentials to advance our understanding of human behavior, and thereby can act as a predictor of human behavior (2). Behaviors and actions of humans are influenced by their mindset of ethical principles and values​​ (3). Considering the link between MI and human behaviors, humans exhibit and creates a system of principles and rules to direct them in distinguishing right from wrong (4). People with high MI levels usually evaluate compatibility of their functionality with ethical principles, leading to enhancement of their commitment and responsibility as well as to improvement of individual and group efficiency (3). 

According to Lennick and Kiel (5), MI involves four principles of integrity, responsibility, compassion, and forgiveness. The principle of integrity is acting on and standing for what we know as right principles, values, and beliefs, thereby creating harmony between words and actions. The principle of responsibility emphasizes on acting consciously, being accountable for our mistakes and failures, accepting responsibility for consequences of our actions as well as being committed to help and serve others. The principle of compassion focuses on respecting to, paying attention to as well as actively and persistently caring for others. The principle of forgiveness advocates flexibility regarding human mistakes and failures considering that human is error-prone and fallible and contains imperfections (5).

MI, as a compass for our actions, leads our other intelligence types (e.g., social, emotional, and cognitive) toward conducting worthwhile actions (6). Individuals with high MI level do the right actions consistent with their values ​​and beliefs, thereby linking their actions with ethics (3).

MI is not inherent and is learned through nurturing, teaching, training, and modeling (i.e., ethics-observing social environment is essential in moral development). Higher education institutions such as universities and colleges are among the most important social environments. In addition to transferring advanced-level knowledge and competencies to students, universities promote social, ethical and cultural values, enhance individual and social skills as well as stimulate personality, emotional, behavioral, intellectual, and moral development (7). Development of moral characteristics (e.g., empathy, self-control, respect, kindness, conscientious action, patience, fairness) in students and their moral growth is important (5) because today’s students will be tomorrow’s professionals of the country. 

Development of MI and moral characteristics among medical sciences’ students, who will work in healthcare and clinical centers in future, will improve the quality of care provided to patients, thereby improving health. Medical students with high MI level can minimize potential risks through careful consideration of their actions’ consequences in the workplace. Since they act according to ethical principles, they perform well both at individual level and in teamwork (5). Considering the importance of MI level, this study aimed to investigate predictor components of MI in SBMU students based on the Lennick, Kiel and Jordan model (5).

## Method

This descriptive-analytical study was performed on SBMU students after receiving the code of ethics (IR.SBMU.PHNS.REC 1397.0999) from the university. Initially, faculties of SBMU were considered as clusters. Then, final samples were selected from each faculty based on the number of students qualified for this study (SBMU students who completed at least one semester at the time of this research) using available sampling method. To observe research ethics obligations, consent of participants was initially received, and their information were kept confidential.

The standard questionnaire of Lennick and Kiel (2011) was used to collect data (5). The questionnaire consists of 40 questions for four components: integrity (10 questions), responsibility (10 questions), forgiveness (10 questions), and compassion (10 questions).

The method used to score this questionnaire was on a five-point Likert (always, often, sometimes, rarely, never). Score range of each participant, ranging from 40 to 200, divided by two to have our target final scores, ranging from 20 and 100. Respectively, score ranges of 90-100, 80-89 ,70-79, 60-69 and 68-0 are indicators of excellent, good, above average, average and poor levels. 

Martin and Austin. initially established validity and reliability of the questionnaire (8). Araste et al. translated the questionnaire from English to Persian, localized it, and established its validity and reliability; Cronbach's alpha reliability was confirmed by Araste et al. and it was shown to be 0.897 (9). The collected data were analyzed using descriptive and analytical statistics by SPSS 21 software.

## Results

From 322 SBMU students participated in the study, 180 were female (55.9%) and 142 were male (44.1%), respectively. The mean age of participants was 22.30±2.69 years, and their age ranged from 18 to 34.

Respectively, students of medical faculty and rehabilitation faculty with overall MI scores of 73.98±6.97 and 70.40±7.45 had the highest mean and the lowest mean. The mean was 72.48±7.28 and 71.28±7.49 for female and male participants’ MI scores, respectively. Statistical tests showed no significant difference between MI scores of female and male genders (*P*=0.14). In addition, no significant correlation was found between variables of age and field of study regarding MI score (*P*<0.05).

The results showed that the mean of MI components’ score was 72.05±7.31, where the highest mean and lowest mean were attributed to the forgiveness and compassion components with scores of 18.49±2.13 and 16.97±2.40, respectively (Table 1).

**Table 1:** Mean and standard deviation of MI’s different components based on Lennick and Kiel’s models

Components	Possible Range	Mean	Standard deviation
Integrity	10-25	18.22	2/31
Responsibility	10-25	18.36	2/17
Forgiveness	10-25	18.49	2/13
Compassion	10-25	16.97	2/40
Total mean	40-100	72.05	7/31

To inspect the most important predictor components of MI in participants, researchers firstly investigated the variables having a significant correlation with MI (*P*≤0.05) based on Pearson correlation test as depicted in Table 2, and then examined them through regression analysis. 

**Table 2:** Correlation coefficient matrix of elements

Components	MI	Integrity	Responsibility	Forgiveness	Compassion
MI	1				
Integrity	**0.812	1			
Responsibility	**0.858	*0.641	1		
Forgiveness	**0.878	**0.637	*0.720	1	
Compassions	**0.810	*0.555	**0.569	**0.658	1

** : Significance at 0.01

**Chart 1:**
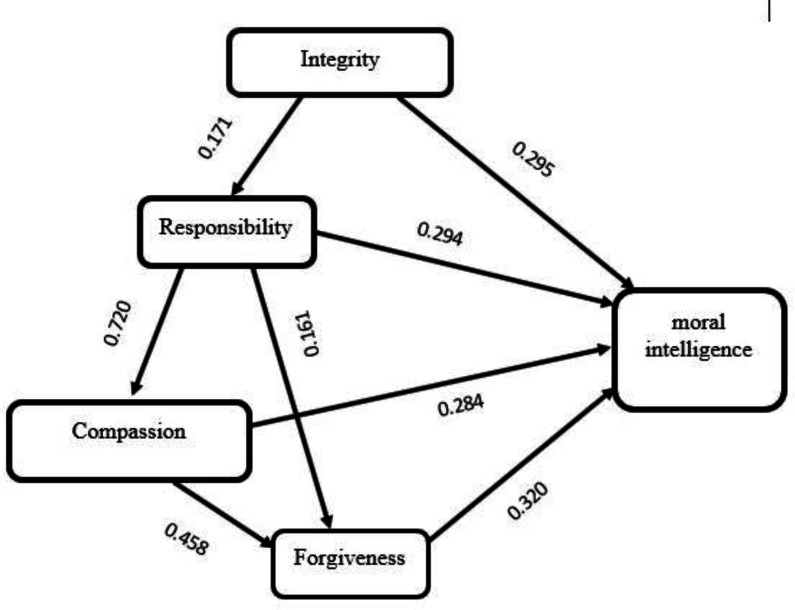
Correlations among the Lennick and Kiel’ predictor components with MI

Results of regression analysis showed that all components directly affect MI (Table 3). The direct and indirect effects among components showed that the most and the least direct effects were related to the forgiveness (R2=0.320) and compassion (R2=0.284) components, respectively. Responsibility component with an overall effect of 0.655 was the strongest predictor component of MI in Lennick and Kiel’s model (Table 3, Chart 1).

**Table 3:** Direct, indirect, and overall effects of Lennick and Kiel’s predictor components on MI

Independent variables	Direct effect	Indirect effect	overall effect	dependent variable
Integrity	0.295	(0.171 × 0.161×0.320) + (0.171 × 0.720 × 0.458×0.320)+(0.171×0.294)+(0.171×0.720 ×0.284)	0.407	Moral Intelligence
Responsibility	0.294	(0.720 × 0.284) + (0.720 × 0.458×0.320) + (0.320 × 0.161)	0.655	
Forgiveness	0.320	-	0.320	
Compassion	0.284	(0.458 × 0.320 )	0.430	

## Discussion

This study’s results showed that the mean of students’ MI total score was 72.05; the integrity, responsibility, forgiveness, and compassion components had scores of 18.22, 18.36, 18.49, and 16.97, respectively.

Rucinski and Bauch showed that the MI level was different in two genders (10); however, Wimalasiri reported that gender had no effect on MI (11). In this study, no significant difference was observed between the two genders’ mean of MI score, and no significant correlation was found between variables of the age and field of study.

To assess MI, Raisi et al. conducted a research in Qom University of Medical Sciences on 210 midwifery-nursing students, using standard Lennick and Kiel’s questionnaire and 5-point Likert scale; the overall mean of students’ MI score was 73.2, indicating that students’ MI level was good (12)

Psychological, social, and hereditary factors can also affect the MI level, and hence these factors may be the cause of differences observed in correlations among demographic characteristics and MI in previous studies. 

Jahaniyan et al. at Kharazmi University conducted a research on 220 post-graduate students in educational sciences using 40-item Lennick and Kiel questionnaire. In this study, students' MI was above average. Statistical analysis results were indicative of a positive and significant correlation between age and MI. In addition, significant differences were observed in MI levels of students majoring in different disciplines (13); biochemistry and anesthesiology disciplines had the highest and the lowest overall MI scores, respectively. Significant differences in MI scores of diverse disciplines indicated the effect of educational curricula as well as the effect of the program and educational environment on student’s MI development. Therefore, the effect of curricula on MI and the influence of MI on professional performance show the role of education on students’ moral and professional development as well as emphasize on the need for efforts to make maximum use of these aspects of education. 

Bayattork et al. conducted a cross-sectional study on 214 medical and nursing students using the 40-item Lennick and Kiel questionnaire. The mean of MI scores in medical and nursing students were 74.07 and 76.44, respectively. Their results were indicative of statistically significant difference between the MI scores’ mean for medical students and nursing students, respectively. In addition, a significant difference was observed between the MI scores of freshmen and senior students. Compared to other MI components, the score of integrity was significantly higher in medical students; and, MI of the students was good (14). 

Bakhtiari and Soleimani at Urmia University conducted a study on MI level of two categories of 20 academic cheating students and 20 non-academic cheating students. The results showed no significant difference between the two groups regarding compassion component; however, for other three components, the scores were lower in academic cheating students (15).

Zerrati et al. in 2014 conducted a research on 359 students of Tehran, Baqiyatallah, Shahed and Shahid Beheshti universities using Lennick and Kiel’s 40-item questionnaire. The results showed that students’ MI was average and below average. A significant correlation was observed among MI level, marital status, and education level factor. The highest and lowest mean score were related to integrity (60.76) and forgiveness (15.15) components, respectively (16). In our study, however, the responsibility component had the highest score because participants believed that they were accountable for their choices’ consequences and actions. According to Lennick and Kiel, humans are responsible for improving each other's lives because all element of the whole world are interdependent (9). In Lennick and Kiel’s view, compassion is more about collective interest than individual interest (5). Thus, in this study, the lowest score on compassion component indicate that the students tend to prioritize personal benefit to collective benefit. 

 Ghaffari et al. conducted a study on medical students of Azad and Public Universities in 2014. Their results showed a positive and direct correlation between students’ academic performance and variables of social intelligence, compassion, responsibility, forgiveness, and integrity (17).

The limitations of this study are as follows: First, cross-sectional studies cannot show the causality, and changes in behaviors resulting from changes in MI level occur over time. Second, as a limitation of self-reporting tools, controlling of all the research variables (e.g., accuracy of answer and bias) and answering to the questions (i.e., completing the questionnaire) cannot be simultaneous.

## Conclusion

A satisfactory level of moral intelligence in students majoring in different disciplines of medical sciences can strengthen the stimulating and effective role of universities in students’ moral development. Since today’s students of medical sciences field will be tomorrow’s medical and health care professionals, upgrading of moral intelligence level in these students enhances their moral responsibility through setting out strong ethical principles to follow and the quality of care that they will provide to patients, thereby improving health.

## Notes:

**Citation to this article:**

Mohammadi M, Mohammadi S, Mehri A, Bagheri Mazraeh F. Investigation of moral intelligence’s predictive components in students of Shahid Beheshti university of medical sciences (SBMU). J Med Ethics Hist Med. 2020; 13: 13.
